# Editorial: Cardiovascular diseases related to diabetes and obesity, volume III

**DOI:** 10.3389/fendo.2024.1381446

**Published:** 2024-02-13

**Authors:** Ying Xin, Jamie Young Wise, Mohanraj Rajesh, Lu Cai

**Affiliations:** ^1^Key Laboratory of Pathobiology, Ministry of Education, Jilin University, Changchun, China; ^2^Department of Pharmacology and Toxicology, University of Louisville School of Medicine, Louisville, KY, United States; ^3^Department of Pharmacology and Therapeutics, College of Medicine and Health Sciences, United Arab Emirates University, Al Ain, United Arab Emirates; ^4^Pediatric Research Institute, Department of Pediatrics, The University of Louisville School of Medicine, Louisville, KY, United States

**Keywords:** metabolic syndrome, obesity, diabetes, cardiovascular disease, CVD biomarkers

Obesity is a chronic, multifactorial metabolic disease responsible for over 4 million deaths each year (1). Globally, the population of obese people is expected to reach more than 1 billion by 2030, accounting for 20% of the total adult population (2). An interaction of genetic, environmental and lifestyle factors (i.e., increased consumption of high-energy food, growth of sitting work patterns, and decreased intensity of physical activity) all contribute to the perpetual rise of disease prevalence. Obesity significantly increases the incidence of many metabolic diseases, and is the primary cause of the global epidemics of diabetes mellitus (DM), hypertension, and cardiovascular disease (CVD) (3). Several cohort studies have also confirmed that obesity is associated with an increase in the mortality from CVD (4). Existing medications fail to cure CVD associated with obesity, DM, and metabolic syndrome, although lifestyle changes, balanced diets, and exercise are available to prevent or delay the complications. Therefore, it is crucial to explore the pathogenesis and early screening approaches of obesity and DM-associated CVD for more effective interventions. This Research Topic developed the third volume, collected ten publications, including 8 clinical studies and 2 reviews, all which have strengthened the understanding of the pathogenesis and early detection of these chronic diseases, providing a platform for the creation of more individualized therapeutic regimens.

CVD is a major contributor to the mortality in the global population; therefore, it is essential to explore effective screening measures to identify high-risk individuals for early intervention. As one of the common pathogenic mechanisms and characteristics of CVD, DM and obesity, insulin resistance (IR) has the potential to be used as an early indicator of disease. However, the application of homeostasis model assessment IR (HOMA-IR), the common assessment approach for measuring IR, is restricted due to its inconvenience and cost. Therefore, Wang et al. performed a prospective cohort study that included 4,712 participants in Xinjiang to estimate the relevance of the metabolic IR score (METS-IR) and triglyceride-glucose (TyG) index, two alternative markers of IR, with the cumulative morbidity of CVD. They revealed that elevated baseline METS-IR and TyG indices were independent risk factors for new-onset CVD, and their trajectory patterns were linked to an increased risk of CVD. Therefore, both METS-IR and TyG index are predicted as early effective assessments for CVD identification in large-scale epidemiological surveys. Furthermore, IR may correlate with the high risk of heart failure (HF) in patients with DM or prediabetes, and recently the triglyceride glucose-body mass index (TyG-BMI) was reported to predict IR better than the TyG index. Thereby, Yang et al. conducted a cross-sectional study involving 7,472 participants, including 329 patients with HF, to investigate the relationship between TyG-BMI and HF. Their study revealed that DM and prediabetes participants with high TyG-BMI were more susceptible to HF, therefore indicating an urgent need to develop strategies to reduce TyG-BMI for HF prevention.

Glycemic variability (GV) refers to fluctuations in glucose levels and has been recognized as a critical risk factor for cardiovascular events, such as HF, diabetic cardiovascular complications, and hypertension. To assess the impact of GV on myocardial recovery in failing hearts and onset of hypertension, the following clinical studies were performed. Among HF patients, a substantial proportion whose ejection fraction (EF) was improved after adherence to guideline-directed medications, is described as HFimpEF, showing better prognosis. A cohort study in 591 consecutive HF patients with a reduced EF (HFrEF, EF ≤40%), hospitalized between 2013 and 2020, was conducted by Yang et al. Duplicate echocardiograms were taken at baseline and after 12 months. Multivariate analysis showed that long-term GV was independently associated with the compromised development of HFimpEF irrespective of glycemic levels. Another large prospective cohort study performed by Huang et al. assessed the correlation of HbA1c, an indicator of GV, with hypertension. This study enrolled a total of 4,074 participants with DM combined with or without hypertension from the China Health and Nutrition Survey (CHNS). Huang et al. (2023) reported a linear and positive correlation between HbA1c and hypertension. These two studies suggest that more steady control of blood glucose is necessary to decrease hypertension and promote cardiac functional recovery in HF patients.

Diabetic cardiomyopathy (DCM), a diabetes-induced microvascular complication, is manifested by myocardial remodeling, diastolic and systolic dysfunction, and poor prognosis, which can ultimately result in clinical HF. Therefore, to improve early assessment of cardiac impairment in DM, Cao et al. designed a study to evaluate left ventricular (LV) function with a non-invasive myocardial work technique. They recruited 67 type 2 diabetes mellitus (T2DM) patients and 28 healthy controls in the study. The results demonstrated that the peak strain dispersion (PSD), global wasted work (GWW) and global work efficiency (GWE) were more sensitive for diagnosing subclinical LV dysfunction among patients with only a small number of changes in diastolic function parameters, while the ejection fraction remained normal. Discordant LV myocardial strain may contribute to the increased GWW and decreased GWE. These findings are very informative, since the uncoordinated LV myocardial strain and abnormal GWW and GWE obtained by this non-invasive technique may serve as early indicators for DCM or other cardiac impairment in T2DM patients. Furthermore, Zhao et al. summarized the DCM literature from the last 40 years, discussing the overall view of DCM, stages of progression, potential clinical indicators, screening and diagnosis criteria, and clinical treatment. In this review, the authors specified five specific spatio-temporal models of DCM and briefly summarized their pathological mechanisms, as well as proposed the hypothesis that a subclinical hyperfunction may be present in the ultra-early stages of DCM. However, more observational clinical trials should be conducted to improve and to explore available metrics and effective drugs for DCM.

Emerging evidence highlighted that visceral adipose tissue might regulate the cardiovascular and metabolic systems through several pathways. Patients with metabolic syndrome (MetS) are considered at high risk for developing CVD, and visceral obesity is the most evident clinical feature of MetS. Chinese Visceral Adiposity Index (CVAI) is utilized for assessing dysfunction of visceral fat and outperforms other indicators in the diagnosis of DM and hypertension among the Chinese population. Given the impact of visceral fat dysfunction on cardiovascular risk, Liu et al. carried out a study aimed at exploring the relationship between CVAI and stroke risk in MetS patients. With 6,732 individuals meeting the criteria for MetS in the Hunan province and a 2-year follow-up, it was found that CVAI showed an independent positive correlation with incident stroke in MetS patients. There is a reduced risk of stroke for MetS patients when the CVAI was below 110.91. Meanwhile, previous studies have found epicardial fat thickness (EFT) isindependently correlated with subclinical carotid atherosclerosis (SCCA), and a recent study showed that perirenal adipose tissue also plays an important role in SCCA. Therefore, Guo et al. undertook a study of 670 T2DM participants to investigate the association between perirenal fat thickness (PrFT) and SCCA for the identification of the novel risk factors. The findings confirmed that PrFT was independently related to carotid intima-to-media thickness (cIMT), plaques, and SCCA. Furthermore, subgroup analysis after stratification for age, sex, smoking, hypertension, and body mass index, also indicated a correlation between PrFT and SCCA. Accordingly, T2DM or MetS with the excessive accumulation of visceral fat, including perirenal fat, ought to attract more attention. In compliance with our call for studies on natural products with potential application in individuals with Mets, a systematic review and meta-analysis of randomized controlled trials was conducted by Qiu et al. to evaluate the efficacy and safety of curcumin on metabolism, inflammation, and oxidative stress among patients with MetS. A total of 785 participants from 13 randomized controlled trials were included, with intervention durations ranging from 4 to 12 weeks. This study confirmed that curcumin improved MetS-related metrics, including high-density lipoprotein cholesterol, inflammatory cytokine levels (i.e., tumor necrosis factor-a and C-reactive protein), and oxidative stress (indicated by MDA). Nevertheless, more studies are needed to confirm these results due to the limited and heterogeneous evidence included.

In addition to obesity and DCM, coronavirus disease 2019 (COVID-19) has emerged as a high risk factor for DM and CVD. In fact, COVID-19 may even be a greater risk factor than obesity and DCM. To evaluate the relation between COVID-19, incidence of CVD, and all-cause mortality among patients with DM, Jung and Choi conducted a study included 16,779 COVID-19 patients and 16,779 matched controls between 2017 and 2021. The results showed that DM patients with COVID-19 experienced higher morbidity and mortality from CVD, coronary heart disease, and stroke compared to uninfected DM patients, which indicates that more prevention and management should be given to DM and MetS patients with COVID-19.

## Author contributions

YX: Conceptualization, Writing – original draft, Writing – review & editing. JW: Writing – review & editing. MR: Writing – review & editing. LC: Conceptualization, Writing – original draft, Writing – review & editing.
